# Shape Memory Graphene with Ultrahigh Specific Energy Dissipation

**DOI:** 10.1002/advs.202508910

**Published:** 2025-08-30

**Authors:** Duc Tam Ho, Udo Schwingenschlögl

**Affiliations:** ^1^ Department of Mechanical and Construction Engineering Northumbria University Newcastle upon Tyne NE1 8ST UK; ^2^ Physical Science and Engineering Division King Abdullah University of Science and Technology (KAUST) Thuwal 23955‐6900 Saudi Arabia

**Keywords:** graphene, molecular dynamics, nano‐architected material, shape memory effect

## Abstract

The properties of architected materials, such as origami, kirigami, and lattice materials, are determined by the intrinsic properties of the base material and the designed architecture. It is shown that van der Waals interaction between the elements of the architecture is critical for the mechanical properties of nano‐architected triangular graphene. Molecular dynamics simulations demonstrate that interplay between the flexibility and high Young's modulus of graphene, the designed architecture, and the van der Waals interaction between graphene ligaments results in a shape memory effect with a specific energy dissipation of more than 25 times that of the well‐known shape memory alloy NiTi. A shape memory effect with ultrahigh specific energy dissipation is ideal for vibration control and reusable impact absorption devices.

## Graphical abstract

Molecular dynamics simulations are used to show that van der Waals interaction is critical for the mechanical properties of nano‐architected triangular graphene. Interplay between the flexibility and high Young's modulus of graphene, the designed architecture, and the van der Waal interaction between graphene ligaments results in a shape memory effect with a specific energy dissipation of more than 25 times that of the well‐known shape memory alloy NiTi.

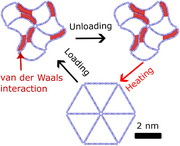

## 1 Introduction

Shape memory materials have the ability to deform under mechanical loading, retain the deformation after unloading, and return to their original shape upon exposure to external stimuli, such as temperature, a magnetic field, or an electric field. This shape memory effect enables commercial applications in different fields, including aerospace technology,^[^ ^1^ ^]^ medical devices,^[^ ^2^ ^]^ vibration control,^[^ ^3^ ^]^ automotive technology,^[^ ^4^ ^]^ actuation,^[^ ^5^ ^]^ and reusable energy dissipation.^[^ ^6^ ^]^ It is observed in shape memory alloys, such as CuAlZn^[^ ^7^ ^]^ and NiTi,^[^ ^8^ ^]^ shape memory polymers, such as polyurethane^[^ ^9^ ^]^ and polylactic acid,^[^ ^10^ ^]^ and shape memory ceramics, such as ZrO_2_,^[^ ^11^ ^]^ primarily due to non‐diffusive phase transformations. The shape memory effect is also found in some architected materials (artificial materials designed to exploit interplay between a base material and a specific architecture to achieve enhanced properties), such as bi‐materials with mismatched temperature‐dependent elastic moduli,^[^ ^12^, ^13^ ^]^ due to snapping responses. Unlike failure mechanisms, such as plasticity in metals and fragmentation in ceramics, the phase transformation and snapping response are reversible, making them ideal for reusable impact absorption devices, for example.

The existing shape memory materials have limitations in their specific energy dissipation. For instance, NiTi has a specific energy dissipation of less than 1.6 J g^−1^,^[^ ^14^ ^]^ which is among the highest values for shape memory materials. Therefore, search for novel shape memory materials and metamaterials with higher specific energy dissipation is important. In this study, we introduce nano‐architected triangular graphene as a shape memory material with ultrahigh specific energy dissipation, an order of magnitude higher than those of the existing shape memory alloys. The material potentially can be synthesized similar to nano‐architected honeycomb graphene by carbon sublimation,^[^ ^15^ ^]^ as both the growth rate and catalysts are effective measures to control the synthesis of carbon allotropes.^[^ ^16^, ^17^ ^]^ Using molecular dynamics (MD) simulations, we demonstrate for nano‐architected triangular graphene a specific energy dissipation of 42.5 J g^−1^. Previously, the stability of nano‐architected graphene foams has been confirmed by density functional theory^[^ ^18^, ^19^ ^]^ and experiment.^[^ ^15^ ^]^ We show that the shape memory effect with ultrahigh specific energy dissipation arises from interplay between the flexibility and high Young's modulus of graphene, the designed architecture, and the van der Waals interaction between graphene ligaments.

## 2 Results and Discussion

**Figure 1:**
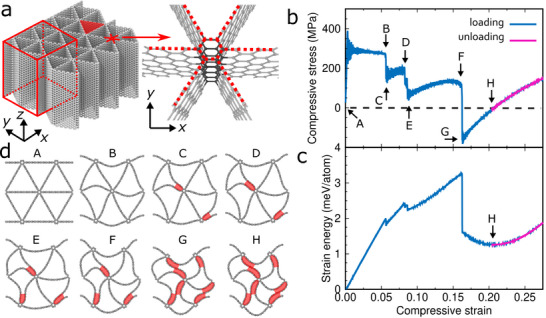
a) Response of nano‐architected triangular graphene (unit cell marked) under uniaxial compressive stress in the *x*‐direction, b) stress–strain curve, c) strain energy‐strain curve, and d) structures at the strains marked in (b). The van der Waals interaction acts as adhesive force, holding the graphene ligaments together after unloading.

We consider nano‐architected triangular graphene with a pore area of 276 Å^2^ (**Figure** **1**a) and junctions consisting of honeycomb rings stacked in the *z*‐direction, resulting in sp^2^ hybridization (Figure 1a). We examine by MD simulations the mechanical response at 1 K for a unit cell with periodic boundary conditions in all directions under uniaxial compressive stress in the *x*‐direction using a strain rate of 10^7^ s^−1^ (see the Simulation Methods section for details). We find initially linear elasticity followed by a stress plateau up to 5.6% strain, which corresponds to buckling of the graphene ligaments (point B in Figure 1b,d). A stress plateau also has been observed in the corresponding macro‐architected material and has been explained by buckling of the ligaments.^[^ ^20^ ^]^ When the strain increases to 16.5%, the stress drops several times instead of remaining constant as in the case of the macro‐architected material (points C to G in Figure 1b,d). No defects, such as dislocations and cracks, are observed at the stress drops. Instead, each stress drop corresponds to a snapping of two adjacent graphene ligaments, causing the distance between them to decrease to less than 3.5 Å in the areas colored in red in Figure 1d, which is close to the spacing of adjacent graphene layers in graphite (3.34 Å). When the stress becomes negative at point G there is a maximum of 7 such areas. The stress then increases to zero (point H in Figure 1b), where a local minimum of the strain energy indicates a stable phase (Figure 1c). The nano‐architected triangular graphene thus experiences a phase transformation from the straight‐edge triangular phase at point A to a wavy‐edge triangular phase at point H due to the mechanical loading. This phase transformation is essential for the shape memory effect, as it enables the material to retain the wavy‐edge triangular phase after unloading.

**Figure 2:**
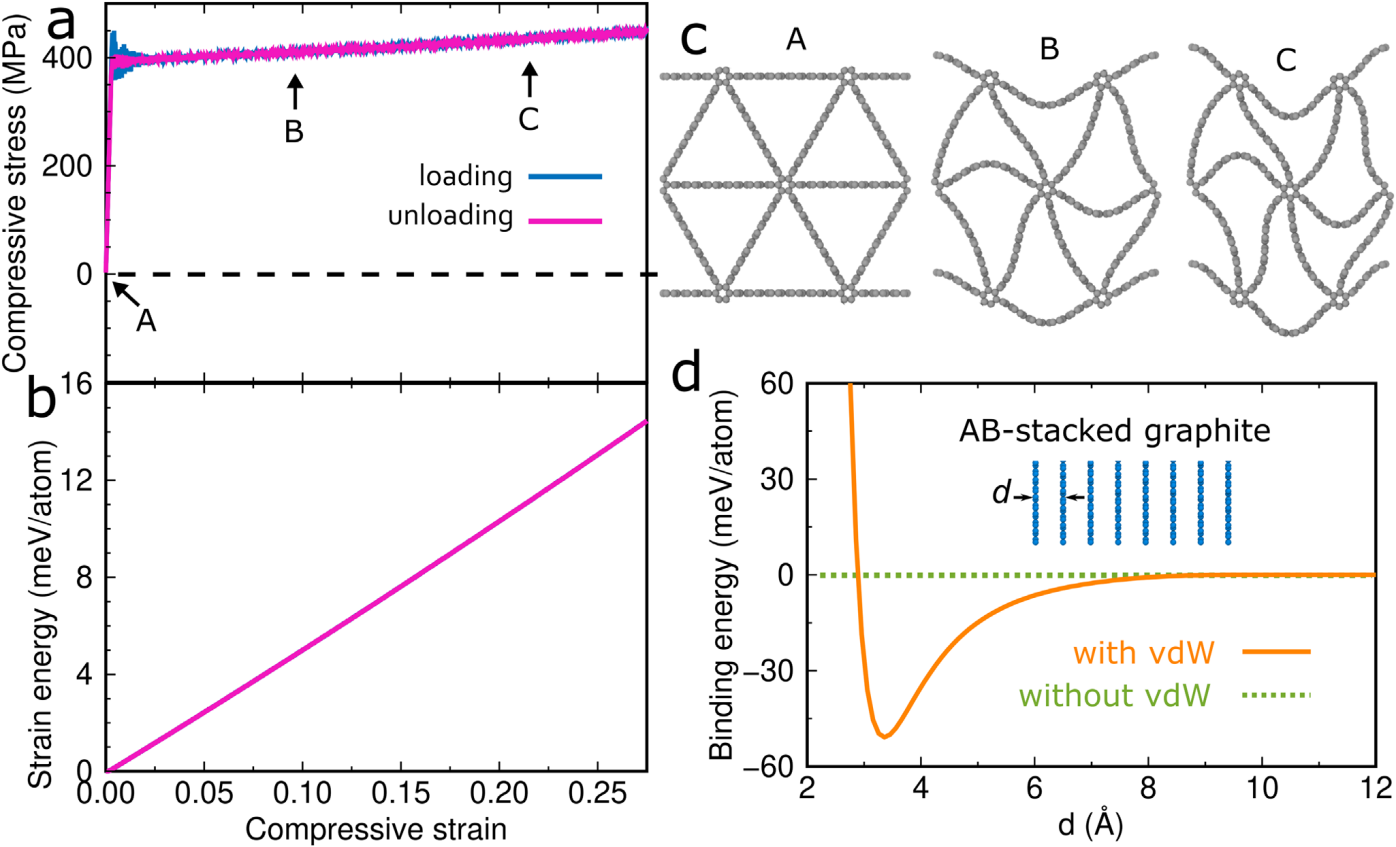
a) Stress–strain curve and b) strain energy‐strain curve of nano‐architected triangular graphene under uniaxial compressive stress in the *x*‐direction without van der Waals interaction, c) structures at the strains marked in (a), and d) binding energy as a function of the distance between the graphene layers of AB‐stacked graphite with and without van der Waals interaction.

The key difference between nano‐architected triangular graphene and the corresponding macro‐architected material is the van der Waals interaction between the graphene ligaments. To understand its effect on the phase transformation, we switch it off in the MD simulations, which results in the same deformation behavior as for the corresponding macro‐architected material,^[^ ^20^ ^]^ specifically a single stress plateau without stress drops (**Figure** **2**a,b), no local minimum of the strain energy, and identical loading and unloading curves, i.e., no phase transformation. This shows that the van der Waals interaction enables the phase transformation, distinguishing the mechanical behavior of nano‐architected triangular graphene from that of the corresponding macro‐architected material. The binding energy due to the van der Waals interaction between the graphene ligaments at 0 K can be calculated by molecular statics calculations for AB‐stacked graphite (see the Simulation Methods section for details). We obtain a value of 50.5 meV per atom (Figure 2d), which is identical to the density functional theory result^[^ ^21^ ^]^ and close to the experiment result of 52.1 meV per atom.^[^ ^22^ ^]^

**Figure 3:**
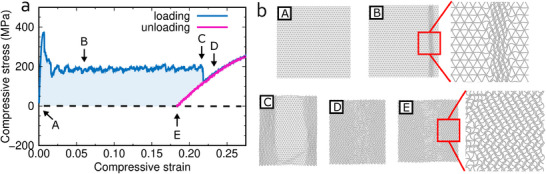
Loading‐unloading process under uniaxial stress in the *x*‐direction for 16 × 18 unit cells with periodic boundary conditions applied in all directions at 300 K: a) stress–strain curves and b) structures at the strains marked in (a). Local phase transformation is observed from the stress drop to point C and full phase transformation is observed after point C. The transformed phase persists when more strain is applied and after unloading.

We next investigate the shape memory effect for 16 × 18 unit cells (for which we obtain at an increased strain rate of 10^8^ s^−1^ convergence of the results with respect to the cell dimension; Figure S1, Supporting Information) with periodic boundary conditions applied in all directions at 300 K. The buckling under compressive stress is found to occur locally rather than homogeneously, implying that the phase transformation initiates locally and spreads out when the strain increases (with the full phase transformation achieved at 22% strain; **Figure** **3**a,b). This results in a wide stress plateau (from 2% to 22% strain) rather than in distinct stress drops as in the case of the unit cell, resembling the phase transformations of some bi‐stable metamaterials.^[^ ^26^, ^27^, ^28^ ^]^ The transformed phase is retained after unloading, confirming the shape memory effect. The specific energy dissipation (shaded area under the stress–strain curve in Figure 3a per mass) turns out to be 42.5 J g^−1^, which is more than 25 times that of NiTi^[^ ^14^, ^23^ ^]^ and more than 40 times that of standard shape memory polymers^[^ ^24^, ^25^ ^]^ (**Table** **1**). The high Young's modulus of graphene (∼1 TPa^[^ ^29^ ^]^) results in high stress in the plateau region and thereby in the ultrahigh specific energy dissipation. Experimentally, graphene foams show excellent compressibility and damping performance,^[^ ^30^, ^31^, ^32^ ^]^ but they suffer from low Young's modulus (typically below 1 MPa) because of low mass density and low interconnectivity,^[^ ^33^ ^]^ resulting in low specific energy dissipation.^[^ ^34^ ^]^

**Table 1:** Mechanical properties of nano‐architected triangular graphene compared to other shape memory materials.

Material	Young's modulus [GPa]	Plateau stress [MPa]	Specific energy dissipation [J g^−1^]	Ref.
Nano‐architected triangular graphene	108	185	42.5	This work
NiTi	—	—	1.6	[14]
Nano‐grained NiTi	45	350	2.4	[23]
Shape memory polyurea	≪1	12	1.0	[24]
Shape memory polyurethane	≪1	—	0.1	[25]

**Figure 4:**
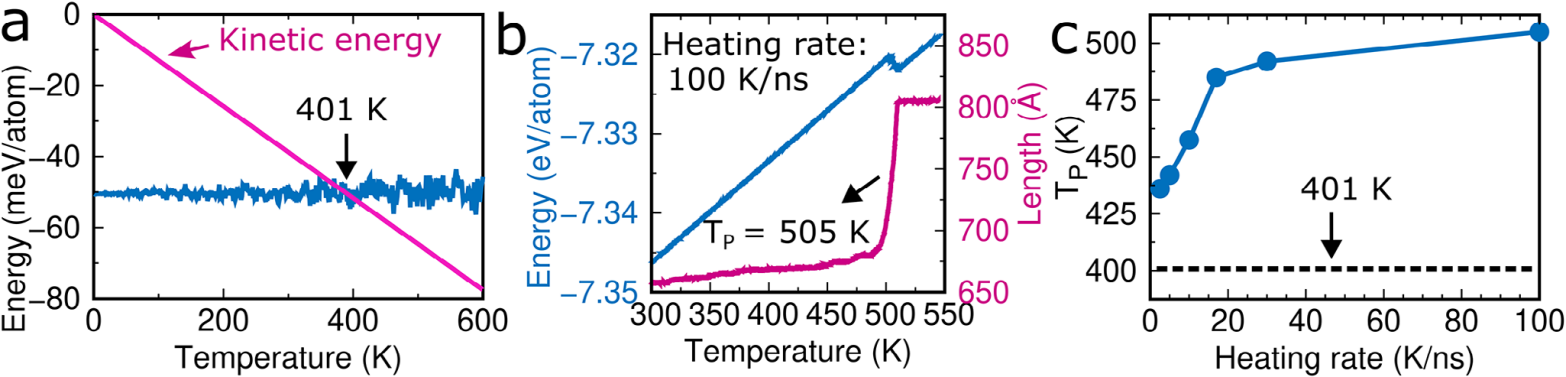
Phase transformation of nano‐architected triangular graphene upon heating. a) Kinetic energy and van der Waals binding energy of graphite as functions of the temperature, b) length in the *x*‐direction and potential energy of the wavy‐edge triangular phase (16 × 18 unit cells) at point E in Figure 3a as functions of the temperature, and c) phase transformation temperature as a function of the heating rate.

A lower limit of the temperature required for recovery of the original phase is given by the temperature at that the kinetic energy and van der Waals binding energy of graphite are equal (thermodynamic driving force). According to **Figure** **4**a, this is the case at 401 K (see the Simulation Methods section for details). To simulate the recovery of the original phase upon heating, we consider the wavy‐edge triangular phase at point E in Figure 3a. When the temperature is increased from 300 to 550 K in 2.5 ns, corresponding to a heating rate of 100 K ns^−1^, the length in the *x*‐direction (due to thermal expansion) and potential energy initially increase (Figure 4b). The length in the *x*‐direction then jumps and the potential energy drops at 505 K due to recovery of the straight‐edge triangular phase. Once the recovery is completed, both quantities increase again; however, the length in the *x*‐direction increases much slower than before, as the material is stiffer in the straight‐edge triangular phase. To investigate the effect of the heating rate, we repeat the simulation with different heating rates from 2.5 to 30 K ns^−1^. The temperature of the phase transformation is found to increase with the heating rate (Figure 4c), being always higher than 401 K, because a phase transformation requires at least the thermodynamic driving force. Impurities and functionalization of nano‐architected triangular graphene can affect the mechanical response and phase transformation. In particular, formation of chemical bonds between the graphene ligaments will shift the phase transformation to higher temperature, whereas steric hindrance of the van der Waals interaction will shift it to lower temperature.

## 3 Conclusion 

In conclusion, MD simulations for nano‐architected triangular graphene demonstrate a shape memory effect with ultrahigh specific energy dissipation. Specifically, combination of the flexibility of graphene with the designed architecture results in micro‐buckling under loading. As van der Waals interaction holds the buckled graphene ligaments together, a phase transformation is induced. The original phase is recovered upon heating, resulting in a shape memory effect. The obtained ultrahigh specific energy dissipation can be attributed to graphene's high Young's modulus. The shape memory effect with ultrahigh specific energy dissipation is a unique property, arising from interplay between the flexibility and high Young's modulus of graphene, the triangular architecture, and the van der Waals interaction. Nano‐architected triangular graphene has great potential in the fields of vibration control and reusable impact absorption devices, for example, where high specific energy dissipation is required. Overall, the van der Waals interaction emerges as novel and highly effective design factor for nano‐architected materials.

## 4 Simulation Methods

The open‐source LAMMPS software^[^ ^35^ ^]^ is employed to perform MD (timestep of 1 fs) and molecular statics simulations, and the OVITO software^[^ ^36^ ^]^ is employed to visualize the results. The temperature and pressure are controlled by a Nosé‐Hoover thermostat^[^ ^37^, ^38^ ^]^ and barostat,^[^ ^39^, ^40^ ^]^ respectively. All the stress components are controlled to be zero in the MD simulations with NPT ensemble except for the loading and unloading processes in which the stress component in the *x*‐direction is not restrained.

MD simulations are used to study the mechanical response. The equilibrium structure of nano‐architected triangular graphene is obtained as follows. The temperature is linearly increased from 0 to 1000 K during 150 ps using an NVT ensemble, then is kept at 1000 K for 150 ps using an NPT ensemble, and then is linearly decreased to 300 K during 300 ps using an NVT ensemble. Afterward, a dynamical relaxation is performed at 300 K for 3000 ps using an NPT ensemble. Compressive strain is applied in the *x*‐direction with a strain rate of ∓10^7^ s^−1^ to simulate the loading and unloading processes using an NPT ensemble. The chosen strain rate balances between accuracy and computational cost: While the critical stress for buckling of the graphene ligaments analytically is $\frac{2n^{2}\pi^{2}D}{\sqrt{3}d^{3}}=330$ MPa with *n* = 1.636, *D* = 0.155 nN, and *d* = 2.42 nm,^[^ ^20^, ^21^ ^]^ we find the stress to fluctuate around 300 MPa, implying that the choice is appropriate.

Molecular statics simulations are used to calculate the binding energy between the graphene layers (distance *d*) of AB‐stacked graphite with initial dimensions of 24.2 Å × 21.0 Å × 40.1 Å and periodic boundary conditions applied in all directions at 0 K as a function of *d*. The structure is relaxed at each value of *d* using the conjugate gradient method with an energy tolerance (relative energy change between successive iterations) of 10^−16^. The *x*‐ and *y*‐dimensions are allowed to change to ensure zero stress in these directions, whereas the *z*‐dimension is fixed. The binding energy is calculated as *E*_*b*_ = *E*_*graphite*_ − *E*_*graphene*_, where *E*_*graphite*_ and *E*_*graphene*_ are the potential energies per atom of graphite and graphene, respectively.

MD simulations are used to calculate the binding energy between the graphene layers of AB‐stacked graphite with initial dimensions of 24.2 Å × 21.0 Å × 40.1 Å and periodic boundary conditions applied in all directions as a function of the temperature. The structure is initially dynamically relaxed at 0.1 K for 50 ps. The temperature is then linearly increased to 600 K during 300 ps and *E*_*graphite*_(*T*) is obtained. The same procedure is adopted for graphene to obtain *E*_*graphene*_(*T*).

MD simulations are used to study the phase transformation upon heating. The temperature is increased from 300 K with heating rates from 2.5 to 100 K/ns until the wavy‐edge triangular phase transforms into the straight‐edge triangular phase.

## Conflict of Interest

The authors declare no conflict of interest.

## Supporting information

Supporting Information

## Data Availability

The data that support the findings of this study are available from the corresponding author upon reasonable request.

## References

[1] J. Mohd Jani , M. Leary , A. Subic , M. A. Gibson , Mater. Des. 2014, 56, 1078.

[2] Y. Xia , Y. He , F. Zhang , Y. Liu , J. Leng , Adv. Mater. 2021, 33, 2000713. 10.1002/adma.202000713 32969090

[3] A. Tabrizikahou , M. Kuczma , M. Łasecka Plura , E. Noroozinejad Farsangi , M. Noori , P. Gardoni , S. Li , Constr. Build. Mater. 2022, 342, 127975.

[4] S. Shreekrishna , R. Nachimuthu , V. S. Nair , J. Intell. Mater. Syst. Struct. 2023, 34, 499.

[5] M.‐S. Kim , J.‐K. Heo , H. Rodrigue , H.‐T. Lee , S. Pané , M.‐W. Han , S.‐H. Ahn , Adv. Mater. 2023, 35, 2208517. 10.1002/adma.202208517 37074738

[6] K. Wang , G. Sun , J. Wang , S. Yao , M. Baghani , Y. Peng , Eng. Struct. 2023, 279, 115626.

[7] E. M. Mazzer , M. R. da Silva , P. Gargarella , J. Mater. Res. 2022, 37, 162.

[8] T. Resendes , P. Freitas Rodrigues , F. Cruz , D. Gatões , V. M. Santos , A. S. Ramos , M. T. Vieira , Prog. Additive Manuf. 2025, 10, 219.

[9] S. Bhatt , R. Pathak , V. D. Punetha , M. Punetha , React. Funct. Polym. 2023, 191, 105678.

[10] F. Fereydoonpour , S. Dezianian , M. Azadi , Polym. Test. 2025, 147, 108802.

[11] N. Zhang , M. Asle Zaeem , Extreme Mech. Lett. 2021, 46, 101301.

[12] Y. Zhang , M. Velay‐Lizancos , D. Restrepo , N. D. Mankame , P. D. Zavattieri , Matter 2021, 4, 1990. 10.1038/s41598-019-48581-8 PMC6715794 31467381

[13] H. Yang , N. D'Ambrosio , P. Liu , D. Pasini , L. Ma , Mater. Today 2023, 66, 36.

[14] J. Cai , S. Mao , Y. Liu , L. Cui , J. Zhang , Z. Zhang , X. Han , Mater. Today Nano 2022, 19, 100238.

[15] N. V. Krainyukova , E. N. Zubarev , Phys. Rev. Lett. 2016, 116, 055501. 26894716 10.1103/PhysRevLett.116.055501

[16] N. Arora , N. N. Sharma , Diamond Relat. Mater. 2014, 50, 135.

[17] G. Churilov , A. Popov , N. Vnukova , A. Dudnik , N. Samoylova , G. Glushenko , Fullerenes, Nanotubes Carbon Nanostruct. 2016, 24, 675.

[18] N. Park , J. Ihm , Phys. Rev. B 2000, 62, 7614.

[19] M. Wu , X. Wu , Y. Pei , Y. Wang , X. C. Zeng , Chem. Commun. 2011, 47, 4406. 10.1039/c0cc05738j 21394348

[20] H. Fan , F. Jin , D. Fang , Mater. Des. 2009, 30, 4136.

[21] D. T. Ho , T. P. N. Nguyen , A. Jangir , U. Schwingenschlögl , Nanoscale Horiz. 2023, 8, 1082. 37255374 10.1039/d2nh00475e

[22] W. Wang , S. Dai , X. Li , J. Yang , D. J. Srolovitz, Q. Zheng , Nat. Commun. 2015, 6, 7853. 26314373 10.1038/ncomms8853 PMC4560750

[23] A. Ahadi , Q. Sun , Acta Mater. 2015, 90, 272.

[24] W. Liu , Y. He , J. Leng , ACS Appl. Mater. Interfaces 2023, 15, 2163. 36571177 10.1021/acsami.2c18489

[25] M. Coccia , E. Farotti , G. Chiappini , T. Bellezze , M. Sasso , J. Intell. Mater. Syst. Struct. 2023, 34, 751.

[26] S. Shan , S. H. Kang , J. R. Raney , P. Wang , L. Fang , F. Candido , J. A. Lewis , K. Bertoldi , Adv. Mater. 2015, 27, 4296. 26088462 10.1002/adma.201501708

[27] D. Restrepo , N. D. Mankame , P. D. Zavattieri , Extreme Mech. Lett. 2015, 4, 52.

[28] S. Yan , L. Wu , Y. Wen , J. Sun , J. Zhou , Responsive Mater. 2025, 3, e20240035.

[29] D. Akinwande , C. J. Brennan , J. S. Bunch , P. Egberts , J. R. Felts , H. Gao , R. Huang , J.‐S. Kim , T. Li , Y. Li , K. M. Liechti , N. Lu , H. S. Park , E. J. Reed , P. Wang , B. I. Yakobson , T. Zhang , Y.‐W. Zhang , Y. Zhou , Y. Zhu , Extreme Mech. Lett. 2017, 13, 42.

[30] A. Nieto , B. Boesl , A. Agarwal , Carbon 2015, 85, 299.

[31] P. Nautiyal , B. Boesl , A. Agarwal , Small 2017, 13, 1603473. 10.1002/smll.201603473 28026152

[32] S. Luo , Y. A. Samad , V. Chan , K. Liao , Matter 2019, 1, 1148.

[33] K. Zhao , T. Zhang , H. Chang , Y. Yang , P. Xiao , H. Zhang , C. Li , C. S. Tiwary , P. M. Ajayan , Y. Chen , Sci. Adv. 2019, 5, eaav2589. 30993202 10.1126/sciadv.aav2589 PMC6461457

[34] L. Qiu , J. Z. Liu , S. L. Y. Chang , Y. Wu , D. Li , Nat. Commun. 2012, 3, 1241. 23212370 10.1038/ncomms2251

[35] S. Plimpton , J. Comp. Phys. 1995, 117, 1.

[36] A. Stukowski , Modell. Simul. Mater. Sci. Eng. 2010, 18, 015012.

[37] S. Nosé , J. Chem. Phys. 1984, 81, 511.

[38] W. G. Hoover , Phys. Rev. A 1985, 31, 1695. 10.1103/physreva.31.1695 9895674

[39] G. J. Martyna , D. J. Tobias , M. L. Klein , J. Chem. Phys. 1994, 101, 4177.

[40] W. Shinoda , M. Shiga , M. Mikami , Phys. Rev. B 2004, 69, 134103.

