# Supplementary material for: Shape Memory Graphene with Ultrahigh Specific Energy Dissipation
Source: Adv Sci (Weinh). 2025 Aug 30;12(40):e08910. doi: 10.1002/advs.202508910 (PMC12561352; doi:10.1002/advs.202508910)
Supplement: Supplementary file 1 — Supporting Information [file ADVS-12-e08910-s001.pdf]

Supporting Information:
Shape memory graphene with ultrahigh specific energy dissipation

Duc Tam Ho[a], Udo Schwingenschlögl[b]

[a]*Department of Mechanical and Construction Engineering, Northumbria University, Newcastle upon Tyne NE1 8ST, United Kingdom.*

[b]*Physical Science and Engineering Division, King Abdullah University of Science and Technology (KAUST), Thuwal 23955-6900, Saudi Arabia.*

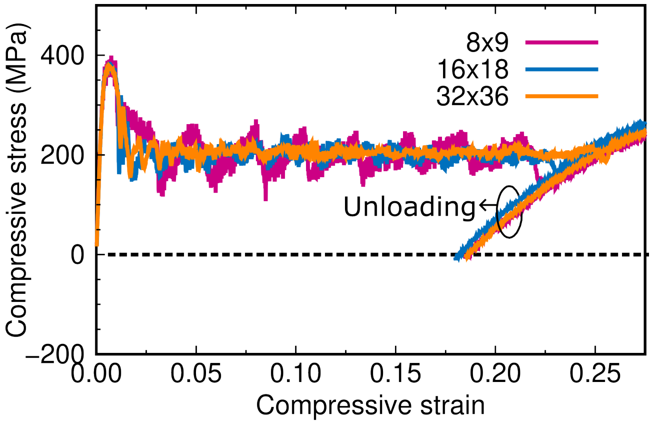

Figure 1: Stress-strain curves at 300 K for different cell dimensions with periodic boundary conditions applied in all directions.
